# SAMuS: Service-Oriented Architecture for Multisensor Surveillance in Smart Homes

**DOI:** 10.1155/2014/150696

**Published:** 2014-03-04

**Authors:** Sofie Van Hoecke, Ruben Verborgh, Davy Van Deursen, Rik Van de Walle

**Affiliations:** Ghent University-iMinds, Multimedia Lab, Gaston Crommenlaan 8 bus 201, 9050 Ghent, Belgium

## Abstract

The design of a service-oriented architecture for multisensor surveillance in smart homes is presented as an integrated solution enabling automatic deployment, dynamic selection, and composition of sensors. Sensors are implemented as Web-connected devices, with a uniform Web API. RESTdesc is used to describe the sensors and a novel solution is presented to automatically compose Web APIs that can be applied with existing Semantic Web reasoners. We evaluated the solution by building a smart Kinect sensor that is able to dynamically switch between IR and RGB and optimizing person detection by incorporating feedback from pressure sensors, as such demonstrating the collaboration among sensors to enhance detection of complex events. The performance results show that the platform scales for many Web APIs as composition time remains limited to a few hundred milliseconds in almost all cases.

## 1. Introduction

As a result of the falling birthrate and the increased life expectancy, the world's population is aging [1]. This aging population and a shift in the burden of illness from acute (infections and injury) to chronic conditions (e.g., asthma, epilepsy, and heart disease) drive up health costs and create a generation of people living with long-term illness and disability. In order to cope with the impact of chronic diseases, disability, or aging, patients are discharged earlier from hospitals, receiving care in their own homes. They have to rely on surveillance services to monitor their health and on assistance when needed. This patient-centered concept of bringing care from the hospital to the patient at home aims to significantly reduce healthcare expenses [2]. Furthermore, the patient-centered concept of living longer independently at home also fulfills the growing social desire for a better quality of life. As “home” for the elderly is a place full of memories where they like to spend their time, equipping this location with advanced electronics and sensors allows them to live independently in their preferred environment.

These so-called “smart homes” are especially equipped for remote monitoring, care delivery, and early detection of health problems, serving especially elderly and people with disabilities. To achieve this, nonobtrusive embedded objects and sensors surround the inhabitants of these smart homes and recognize individual users and their situational context. The sensors either measure simple ambient conditions or capture video related to the environment surrounding the sensor. Examples include temperature sensors, camera monitoring sensors, light sensors, presence and weight sensors, toilet flush sensors, automated switch-offs for dangerous devices such as cookers and stoves, and visitor identification cameras.

Processing the signals from the different sensors reveals some properties about objects located and/or events happening in their vicinity. However, sensors typically exert no effect on each other, which is a suboptimal mode of operation. Each sensor stands on its own, delivering information without taking into account feedback from other neighbor sensors, imposing a lot of restrictions to smart homes. Additionally, optimizing the reliability of information retrieved from single sensors has led to intensive research in the past few years, yet has reached its limits [3]. Therefore, to further improve the support people get from sensor systems in their everyday lives, collaborative gathering and processing of sensor data become necessary. This way, the available information and intelligence of all sensors can be fed back to each of them in order to optimize their functionality. For example, when a visitor needs to be identified, or a demented patient must be monitored, the smart home platform can dynamically switch to infrared video sensors under bad illumination conditions or use another video processing algorithm in response to changes in movement, temperature, or lighting.

The combination of sensor information within a smart home platform is a promising approach in order to enhance the detection and interpretation of advanced events, and also the topic of this paper. Unfortunately, it also comes with a number of problems [4]: by combining sensor data, the amount of available data rapidly increases. Therefore, the following challenges need to be tackled in order to cope with such an amount of data:

- representation of the sensor data: different sensors communicate their results through different protocols and represent their data in different formats, resulting in a huge heterogeneity in terms of sensor data representation;
- finding relevant sensor data: not all sensors can be combined with each other, which implies that we need to investigate which sensor combinations make sense and how this can be expressed;
- performance issues: since smart homes have to deal with a large amount of sensor data, possible performance issues need to be anticipated during the detection and interpretation of events.

Current smart home solutions use different, proprietary, or noncompatible technologies that hinder mass-market development [5–7]. To overcome this issue, the service-oriented architecture for multisensor surveillance in smart homes (SAMuS), presented in this paper, adopts the Internet of Things vision and implements the sensors as Web-connected devices having a uniform Web API. This way, all “things,” that is, sensors, are connected with similar technology, serving as “gold standard” solving current barriers in mass-market adoption of smart homes. Moreover, advanced reasoning and interpretation strategies can be applied to the fusion of the sensor data, allowing combining information between video sensors (cameras) and other nonvideo sensors (such as temperature, sound, and heart rate). This in turn enables an enhanced functionality of the sensors by allowing them to detect complex events that currently remain undetected. As a result, the surveillance and care delivery services within a smart home platform can be significantly improved. For example, for demented patients having difficulties in remembering the steps in doing everyday activities such as washing their hands, the smart home platform will monitor their movements and if the patient does not pick up the soap, it gives instructions or shows video demonstrations. Additionally, the platform can identify visitors, automatically switch off dangerous devices when needed, and log everything in order to reassure the patient and help him recall memories which will lead to long-term retention of memories in the end. The smart home platform also allows the patients to be monitored and supported by caregivers and family remotely.

The remainder of this paper is structured as follows.  Section 2 gives an overview of the related work on smart homes. In Section 3, the SAMuS platform is presented, more specifically the architecture overview, internal design issues, and broker components. In Section 4, the SAMuS platform is evaluated by creating a smart Kinect sensor able to dynamically switch between IR and RGB and optimizing person detection by incorporating feedback from other sensors. Performance results on scalability are presented as well. Finally, we highlight the main conclusions of this work in Section 5.

## 2. Related Work

Smart homes are a long way from maturity. Although under development for decades now, smart homes have barely made it out of the research labs [6–8]. The idea of smart homes comes from the earlier work on home automation focusing on, for example, indoor climate monitoring [9] or minimizing energy consumption [10]. The MavHome project [11] defines the smart home as an intelligent agent supervising and trying to improve the users' life quality, while keeping in mind ecological factors such as decreasing water consumption. MavHome uses CORBA as underlying technology to connect all the software services and data mining to reduce the database size. The Amigo Project [12] and the Service Centric Home [13] aim at the development of middleware that integrates heterogeneous systems and appliances to achieve interoperability between services and devices [8].

Due to the opportunities of combining sensor information within a smart home platform, multisensor surveillance in smart homes has also been the subject of many researches. The ACHE smart house architecture [10] uses basic sensors, limited to switching on/off, temperature readings, and door open/closed values. All sensor values are processed centrally to define occupancy patterns and adapt the environment to improve the inhabitants' comfort. Neural networks are used to predict future states of the home. Also in [14], simple state sensors are used in combination with pattern recognition and classification algorithms based on naive Bayesian network to detect simple activities like toileting and bathing. Regardless of the suboptimal training method used for the activity recognition, it is shown that it is possible to recognize complex actions with simple sensors.

Finally, the Gator Tech Smart House project [15] uses a service-oriented architecture approach to connect all the sensors and actuators in the smart home. The layered architecture is based on OSGi, where the OSGi bundles contain the definitions of services a particular sensor or actuator can offer. The services can be composed into new, more complex services, and an ontology describes every device in the house, ensuring the services use compatible values while communicating.

These solutions illustrate the potential of smart homes and multisensor surveillance. Not surprisingly, numerous companies compete and cooperate to produce devices and sensors that will help consumers achieve longer living independently. However, despite the complexity of the market, a clear concern is emerging that the market will not grow to its full potential if current barriers, as a result of the different and noncompatible technologies used, remain [5]. Therefore, the sensors within the SAMuS architecture are implemented as Web APIs; a general approach, not limited to OSGi or any other (proprietary) technology, allowing combining information between sensors and detecting complex events that currently remain undetected.

## 3. Design of the Multisensor Surveillance Architecture

The aim of this research is to design a platform for multisensory surveillance. A wide range of applications can benefit from combining visual, audio, and other sensor information. Examples are office or airport security and human tracking, fire detection [16], traffic control systems, advanced health care delivery and assistance to elderly (the use case of this paper), and industrial process control and condition monitoring. In these applications, multimedia support has the potential of enhancing the level of information collected, enlarging the range of coverage, and enabling multiresolution views.

In the subsections below, it is explained how the SAMuS platform tackles the challenges that were listed in Section 1. Furthermore, the SAMuS platform also takes into account the following requirements.

1. Human-platform interaction for deploying new sensors should be straightforward, allowing for mass-market adoption of smart homes.
2. Because the system may be deployed in highly flexible environments, on-the-fly addition and removal of components and sensors are preferred.
3. The platform needs to be generic in the sense that it should be possible to plug in new components, independent of implementation languages, operating systems, and hardware.
4. The platform needs to be scalable so it can handle complex sensor collaborations.

### 3.1. Architecture Overview

**Figure 1:**
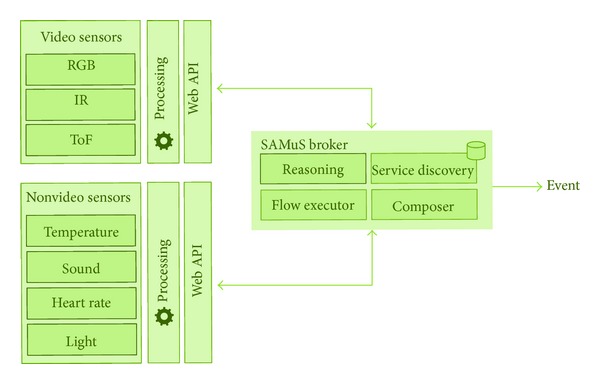
High-level architecture of the SAMuS multisensor surveillance platform.

Figure 1 presents the high level architecture of the SAMuS multisensor surveillance platform that enables integration of sensors (video and nonvideo sensors) using a broker architecture (SAMuS broker) in order to enhance the sensors' functionality and detect advanced events.

The complexity and heterogeneity of these multisensor surveillance systems, where various kinds of sensors need to cooperate while having widely diverse characteristics, directly map onto the service-oriented architecture pattern. These service-oriented architectures benefit from loosely coupled modularity, interoperability, flexibility, and reusability. Therefore, the SAMuS platform is designed based on the principles of service-oriented architectures, wherein all components, including sensors, are implemented as services. More specifically, the SAMuS platform is built around a broker that is able to discover available sensors and services (service discovery component), select and compose sensors (composer and reasoner component), and process the data and sensor flows in order to facilitate multisensor surveillance (flow executor component).

By using the generic concepts of service-oriented computing and brokering, the platform presented in this paper is not restricted to healthcare monitoring in smart homes but acts as a generic communication system in which sensors and services can easily be plugged.

A SAMuS platform prototype as well as some prototype sensors and processing services has been implemented in the iLab.t HomeLab and is currently evaluated. Below is an overview of the main internal design details and the broker components.

### 3.2. Choosing the Service Style

In view of the broad support for Web services, they are perfect for implementing the service-oriented architecture and the integration of heterogeneous software components since applications can easily be distributed and they expose well-defined functionality as a Web service. The Web service technology enables thus the required integration for the SAMuS platform.

Two main Web service style architectures exist, SOAP and REST. The implementation of services in the SAMuS platform is done using the REST style architecture for two reasons. First, REST offers a lightweight communication compared to SOAP [17] as there is no need to build, process, and send long XML files; only the resource is transferred. Second, implementing a Web service on a sensor hardware platform cannot require much computing power. As implementing a REST service only requires HTTP functionality, this is more simpler than implementing a SOAP service on a device with limited capabilities.

### 3.3. Describing the Services

It is impossible to make different sensors and services interoperate if there are no agreements or guidelines on how communication should happen. The coordinating SAMuS platform can only select sensors and services based on their capabilities in presence of a formal description detailing their preconditions and postconditions. Not only for selection, but also for support of dynamic sensor and service composition, the services, its inputs and outputs, and its functionality need to be described in an unambiguously and machine-understandable way.

Different possibilities and standards exist that offer service description. The verbose WSDL is one of the oldest ways to describe services; version 2.0 supports describing REST services. ReLL [18] XML File improves WSDL by adding different representation possibilities to a resource and allowing converting to RDF triples. Finally, RDF [19] also allows describing resources and has a natural compatibility with REST. RDF can also define the semantic value [19] of a resource.

As WSDL does not provide the means to capture the functionality of a service, it cannot offer automatic service discovery at runtime. ReLL is an XML-based standard and thus uses too many resources on the sensors. However, RDF is a universal resource description framework and the best choice for this platform. The RDF language can describe a service in full detail with far less lines than would be needed in an WSDL or ReLL document. Also, with RDF being a framework, different other framework components (such as support for semantics and ontologies) can be used.

RESTdesc [20] is an implementation of the RDF principles, allowing describing services and using semantics and using Notation 3 (N3, [21]). An example RESTdesc service description of a light sensor can be found in Listing 1, using the N3 notation. A GET request to the /lightValue path gives the light sensor's value, representing the lighting condition of the environment. The sensor platform provides this description as a resource that can be discovered by describedBy links.

### 3.4. Service Discovery

In order for the SAMuS platform to be able to discover the available sensors and services, the platform can (i) hardcode the URIs, which is of course not very flexible and maintainable, (ii) use a dedicated service registry that collects all information on the different services but requires changes to be posted to the registry, or—the most flexible and therefore chosen solution—(iii) use a discovery mechanism such as Web linking [22].

A discovery mechanism based on Web linking is implemented in the service discovery component, benefiting from the REST service already being a HTTP server, so only HTTP GET calls need to be performed in order to send the RDF descriptions of the service in return. The drawback of this method, however, is that some entry point to the service need to be advertised to the consumers (such as the base URL), which is not a part of the specification. The discovery algorithm first configures the REST server on the sensor platform by reading a configuration file (e.g., on microSD card). The configuration file specifies the paths that need to be available and the HTTP request types to accept. Once the Web server is set up, the initial root discovery problem is solved by letting the sensor broadcast a “Hello” message containing a hash of the sensor platform's MAC and IP address. The broker's service discovery component picks up the broadcasted messages and does GET or HEAD requests to the according IP address, returning the descriptions and discovering all available options. If no request has been done by the broker within a certain amount of time, the sensor broadcasts the message again.

### 3.5. Service Semantics and Ontology

Semantics provide a uniform way to describe what the data really represents. This is done by referencing to namespaces containing a general understanding of what the data represents and its properties, this way making the data machine-readable without losing the human-readable advantage. This way, data of one sensor can be reused by other types of sensors, reasoning techniques can be applied onto the aggregated sensor data, and advanced semantic service matching and composing techniques can be used to automatically combine different sensors.

Although the value of semantics in service-oriented and broker architectures is recognized, the concept is still challenging and a lot of research remains. Different possibilities and standards exist that offer service semantics. SAWSDL [23] provides WSDL-based semantic annotations and allows adding semantics to a REST service. The annotation mechanism also supports integration of RDF and Web Ontology Language (OWL) statements, providing a simple implementation of ontologies. However, as short RDF-N3 descriptions are chosen to describe the services, SAWSDL is not an option. Semantic Annotation for REST (SAREST) is another annotation language to describe REST services [17]. The problem, however, with SAREST is that semantic descriptions need to be distributed to service consumers. With REST services offering data as a resource, a client must specifically seek a description on a well-defined link, before being able to consume the service properly. A third option is the RDF framework, providing RDF semantics and allowing semantic descriptions to be written in RDF-XML or plain text RDF format. Finally, the OWL-S [24] specification (based on the former DAML-S standard) defines two types of possible expressions: descriptions of rules (defined in SWRL [25]) and description of parameters (defined in either XML or non-XML based markup). With OWL-S descriptions, a lot of data is generated to describe a single simple service. It allows describing complex services and structures in a readable way, but it is hard to set up this description. The RDF framework, on the other hand, allows descriptions to be in non-XML based markup, making it the right choice for our surveillance platform. The ontologies are expressed in OWL. This allows ontological constructs to interact with the semantic RDF descriptions of the services. For instance, the fact that a thermocouple is a subclass of a temperature sensor allows deciding that a thermocouple can be used whenever a temperature sensor is required.

### 3.6. Sensor Collaboration

Semantics describe the measured values of the sensors. When multiple sensors are available in the network, it becomes really interesting when they can cooperate. For example, as healthcare monitoring should work day and night, in all kinds of lighting situations, the value of the environment lighting condition can be used to decide if RGB images or IR images should be used. Using the data from the light sensor, the broker's reasoning module can warn the camera for bad lighting, making the camera change to the IR stream and continue analyzing the images.

However, dynamic composition and integration of sensors and services is challenging and the topic of many research studies as it is still very difficult to put into practical use [26]. Therefore, a novel solution to automated composition of Web APIs is developed and used that does not require new algorithms and tools and can be applied with existing Semantic Web reasoners. Those reasoners can easily incorporate external sources of knowledge such as ontologies or business rules.

To achieve a proper analysis of which services can be composed, a reasoner component is implemented in the broker that takes all the descriptions of the sensors as input and provides a composition that matches a certain goal. In order to do so, the composer module transfers all collected resources (i.e., service descriptions) to the reasoner, along with a goal to achieve. An example goal could be the following:

 
?sensor environment : lightingCondition ?value.

When all the data is processed by the reasoner, the reasoner outputs the path to follow in order to achieve the goal. Since a composition is equivalent to a proof [27], creating a composition that satisfies a goal comes down to generating a proof that supports the goal. Inside this proof, the necessary Web API calls will be incorporated as instantiated rules, containing the various HTTP requests that need to be done.

In our case, the Euler Yap Engine reasoner (EYE) is used, a backward-chaining reasoner enhanced with Euler path detection, as it allows for very fast processing of all the descriptions and generates answers in a performant way. EYE accepts input descriptions in the N3/Turtle format and returns a proof in N3. As N3 contains constructs that are not supported in Turtle, we developed a parser that changes the proof notation so it fits existing Turtle parsers such as Jena (the one used here). Note that the solution is not limited to the EYE reasoner; any reasoner with proof support can be used.

More detailed information on this approach to Web API composition can be found in [27]. The evaluation in Section 4 indicates that proof-based composition is a feasible strategy today.

### 3.7. Executing the Sensor Flows

As already stated, a custom parser was developed to process the generated proof by transforming the formulas so that the Jena parser is able to read the output. The HTTP flow is derived from the formulas describing HTTP requests and the flow executor component then executes the actual requests. To execute the composition, the following algorithm is applied.

1. Find the next Web API call whose required parameters are available, that is, literal values or placeholders that have been filled out in steps 2 and/or 3.
  - If no calls are pending, the composition has been executed and the goal is reached. Go to step 4.
2. Execute the Web API call with the parameter values and augment the state with the retrieved values.
  - If the call fails, a new composition must be generated, starting from the current state but explicitly excluding the failed API as a possibility.
3. Make inferences on the new state using available background knowledge. These inferences can possibly fill out placeholders in pending calls. Go to step 1.
4. The composition has been executed and the goal has been reached (event detected).

## 4. Evaluation

The SAMuS platform design supports a user-friendly sensor deployment: whenever a new sensor needs to be deployed in the SAMuS platform, the only configuration required is the configuration file for the sensor platform as the remainder of deployment is automated, resulting in minimal required user interaction and solving the first requirement. As deploying a new sensor can be done in a dynamic way so that the platform and current services can continue working, the second requirement is also fulfilled. By using a service-oriented architecture and Web APIs, the platform is generic, independent of implementation languages, operating systems and hardware, fulfilling the third requirement. Finally, to test if also the fourth requirement (scalability) is fulfilled, a SAMuS platform prototype as well as some prototype sensors and processing services has been implemented.

The sensor hardware platform, used to connect the actual sensor devices to the SAMuS platform, is based on Netduino Plus devices, which are standard Netduino boards with onchip  .NET framework and on-board debugging capabilities, extended with an on-board Ethernet port and an open-source TCP/IP stack. Implemented sensors are (i) a light sensor, using a simple photoconductive cell changing its resistance according to the amount of light that is perceived (ranging from 1 kOhm for bright light to 10 kOhm for darkness), (ii) a pressure sensor, more specifically a flexiforce pressure or piezoresistive force sensor, changing resistance according to the amount of force applied to the sensor (ranging from 300 kOhm when pressing hard to infinity for no pressure, and (iii) a video sensor, more specifically a Kinect camera providing RGB, IR and depth images. Kinect uses projected speckle patterns in near-infrared light to determine the depth of any given scene. For the Kinect sensor, different algorithms (e.g., for different environment lighting conditions) were implemented to detect faces and eyes.

In order to test platform operation, a smart Kinect sensor is created by coupling three simple sensors (light, pressure, Kinect), this way being able to dynamically switch between IR and RGB and optimizing person detection by incorporating feedback from pressure sensors. The test case is as follows:

- Depending on the light intensity in the room, the Kinect can use IR images instead of RGB images.
- If the Kinect camera was not able to detect a user, the reasoning module can still ask to check the presence of a person for example, if a pressure sensor has reacted. In that case, the camera will try to detect a face or eye in the whole scene.
- Depending on the position of the user and the data from other sensors, the camera can switch between two algorithms to perform a standard, quick detection algorithm or a more advanced algorithm. More advanced algorithm takes too much time to be used for every frame but have a much higher detection rate.

**Figure 2:**
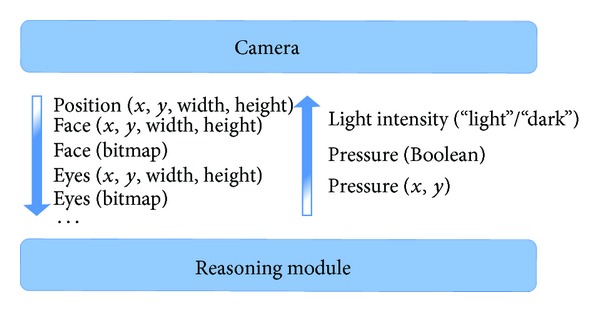
Abstract schema of communication between broker's reasoning module and Kinect camera.

This is just one example of the many possible situations and functionalities that are supported.  Figure 2 shows a schematic overview of communication between the reasoning module of the broker and the Kinect camera for the implemented test case. The reasoning module sends two kinds of data:

- “Light” or “Dark” to allow the camera to switch between IR and RGB;
- The fact that the pressure sensor is pressed or not (true or false) to force the camera to find a person in the image if it is not tracking a user at that time. The position of the pressure sensor (*x*, *y*) when it is pressed can also be of value to increase the performance of the tracking and detection algorithms by reducing the search area.

The camera sends information to the reasoning module on:

- the position of the person in the scene (*x*, *y*, width, height);
- the detected face (bitmap) or the position of the detected face (*x*, *y*, width, height);
- the detected eyes (bitmap) or the positions of the detected eyes (*x*, *y*, width, height).

**Figure 3:**
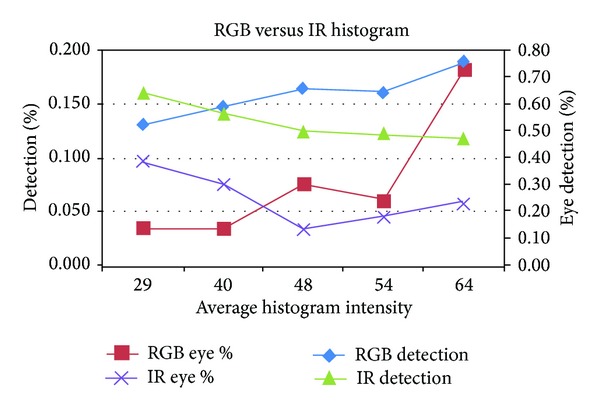
Comparison of hit rates for RGB and IR images in different lighting situations.

Figure 3 shows the resulting recall in RGB and IR for varying lighting situations. As can be seen, the switch between algorithms is best done at an average light intensity between 35 and 45. To be sure to switch on time and still keep the RGB range as big as possible, it was decided to switch at a light intensity value of 40.

This way, once the camera module is turned on, the broadcast is sent and the Kinect waits for responses from the broker. The broadcast listener on the broker accepts the broadcast from the camera and sends a GET request to the camera for more information. Once connected, the camera switches algorithms to stream IR images instead of RGB images (and vice versa) using the threshold of 40 for light intensity. We tested this by (un) covering the light sensor.

As can be seen in Table 1, initial start-up and broadcast procedure takes around ten seconds (i.e. initializing the sensor platform, configuring the web server and sending the broadcast message). The time to process an actual request for sensor data (i.e. read description, dynamic composition, flow execution overhead) is around five seconds. So, operational sensors can be set up in seconds. Video footage of the platform operation can be found at: http://www.youtube.com/watch?v=jS_0YKgpwkU.

In order to prove the scalability of the SAMuS platform (allowing more complex service compositions), we tested the reasoner module's performance when creating proofs with varying length and complexity. The results, presented in Table 2, are achieved for the broker running on a consumer computer (2.66 GHz Intel Core i7, 4 GB RAM). The results in the first column indicate that starting the reasoner introduces an overhead of *≈*50 ms. This includes process starting costs, which are highly machine-dependent. Inspecting Table 2 from left to right, we see the reasoning time increases linearly with the composition length *n* and remains limited to a few hundred milliseconds in almost all cases, fulfilling the fourth requirement. The absolute increase in reasoning time for a higher number of dependencies *d* never crosses 200 ms for small to medium values of *n*, but becomes larger for high *n*.

## 5. Discussion and Conclusion

In this paper the design of a service-oriented architecture for multi-sensor surveillance in smart homes is presented as an integrated solution enabling automatic deployment, dynamic selection and composition of sensors. Sensors can be added with minimal administrator intervention.

The challenges for sensor integration were mentioned in Section 1. These challenges were addressed in the design process in the following way.

By adopting the Internet of Things vision and implementing the sensors as web-connected devices, sensors have a uniform Web API (solving the representation of sensor data challenge). RESTdesc is used to describe the sensors and a novel solution is presented to automatically compose Web APIs that can be applied with existing Semantic Web reasoners (solving the challenge of finding relevant sensor data).

The procedure used for sensor discovery (the broadcast algorithm) allows for a platform independent implementation, independent of the choice of sensor platform (Netduino, Arduino, embedded Linux, etc.). The modular approach of the platform allows for easy alteration of functionality by replacing services and/or modules.

By implementing sensors as web-connected devices with a uniform Web API, barriers of current smart home solutions are reduced. Moreover, thanks to advanced reasoning and interpretation strategies, information between video sensors (cameras) and other non-video sensors (such as temperature, sound, heart rate, etc.) can be combined to detect complex events that remain undetected in current smart home solutions.

The presented solution is not limited to a design study: all platform components are implemented and integrated, allowing an operational multi-sensor surveillance architecture providing sensors to be set up in seconds. We evaluated the solution by building a smart Kinect sensor being able to dynamically switch between IR and RGB and optimizing person detection by incorporating feedback from pressure sensors, illustrating the opportunities of the platform. Additionally, the performance results show that the platform scales for many Web APIs (solving the performance challenge).

Although the proof-of-concept provides a fully operational platform, it is still merely the start. This platform is meant to activate a new way of implementing smart sensor networks in smart homes so scaling up the complexity of sensor interactions is required, allowing more complex service compositions.

## Figures and Tables

**Listing 1:**
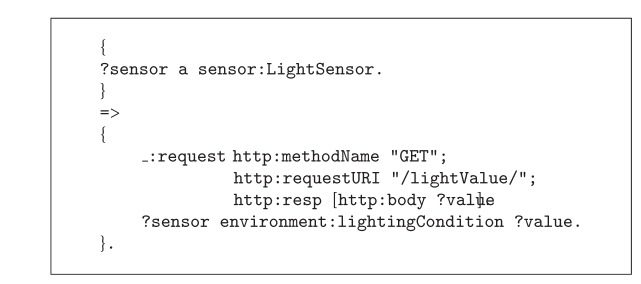
A RESTdesc description of a light sensor.

**Table 1:** Typical durations of configuration steps.

Initial start-up and broadcast procedure	10 s
Read out and save sensor description	4.3 s
Composing the descriptions	See Table 2
Flow execution overhead	50 ms

**Table 2:** The reasoner component manages to create even lengthy compositions in a timely manner (average times of 50 trials).

Number of APIs *n*	2	4	8	16	32	64	128	256	512	1,024
*d* = 1 dependency										
Parsing	53 ms	53 ms	54 ms	55 ms	58 ms	64 ms	78 ms	104 ms	161 ms	266 ms
Reasoning	**2 ms**	**4 ms**	**5 ms**	**7 ms**	**11 ms**	**20 ms**	**43 ms**	**77 ms**	**157 ms**	**391 ms**

Total	55 ms	57 ms	58 ms	62 ms	70 ms	84 ms	121 ms	181 ms	318 ms	657 ms

*d* = 2 dependencies										
Parsing	53 ms	53 ms	59 ms	56 ms	60 ms	67 ms	85 ms	117 ms	184 ms	331 ms
Reasoning	**3 ms**	**6 ms**	**69 ms**	**41 ms**	**45 ms**	**56 ms**	**84 ms**	**174 ms**	**461 ms**	**1,466 ms**

Total	56 ms	59 ms	128 ms	97 ms	104 ms	123 ms	169 ms	292 ms	645 ms	1,797 ms

*d* = 3 dependencies										
Parsing	53 ms	53 ms	68 ms	56 ms	61 ms	70 ms	90 ms	129 ms	208 ms	371 ms
Reasoning	**3 ms**	**12 ms**	**45 ms**	**49 ms**	**61 ms**	**99 ms**	**200 ms**	**544 ms**	**1,639 ms**	**6,493 ms**

Total	57 ms	66 ms	114 ms	105 ms	122 ms	169 ms	290 ms	673 ms	1,847 ms	6,864 ms

## References

[1] Bloom DE, Canning D, Jamison DT (2004). New evidence coupled with a wider perspective suggest sizable economic returns to better health. *Finance and Development*.

[2] Wartena F, Muskens J, Schmitt L Continua: the impact of a personal telehealth ecosystem.

[3] Hu W, Tan T, Wang L, Maybank S (2004). A survey on visual surveillance of object motion and behaviors. *IEEE Transactions on Systems, Man and Cybernetics C*.

[4] Molla MM, Ahamed SI A survey of middleware for sensor network and challenges.

[5] Steigleder C (2013). *Building A smart Home Ecosystem Urgent Need For Standardization*.

[6] Basu D, Moretti G, Gupta GS, Marsland S (2013). Wireless sensor network based smart home: sensor selection, deployment and monitoring. *IEEE Sensors Applications Symposium*.

[7] Anbarasi A, Ishwarya M Design and implementation of smart home using sensor network.

[8] Blumendorf M Building sustainable smart homes.

[9] Ivanov B, Zhelondz O, Borodulkin L, Ruser H Distributed smart sensor system for indoor climate monitoring.

[10] Mozer MC The neural network house: an environment that adapts to its inhabitants.

[11] Das S, Cook DJ Health monitoring in an agent-based smart home.

[12] Georgantas N, Mokhtar SB, Bromberg Y-D The Amigo service architecture for the open networked home environment.

[13] Albayrak S, Blumendorf M, Feuerstack S (2009). Ein framework für ambient assisted living services. *Deutscher Ambient Assisted Living Kongress*.

[14] Tapia EM, Intille SS, Larson K (2004). Activity recognition in the home using simple and ubiquitous sensors. *Lecture Notes in Computer Science*.

[15] Helal S, Mann W, El-Zabadani H, King J, Kaddoura Y, Jansen E (2005). The Gator tech smart house: a programmable pervasive space. *Computer*.

[16] Verstockt S, Van Hoecke S, Tilley N, Lin W (2011). Hot topics in video fire surveillance. *Video Surveillance*.

[17] Gomadam K, Ranabahu A, Sheth A (2010). *Sa-Rest: Semantic Annotation of Web Resources*.

[18] Alarcon R, Wilde E Linking data from restful services.

[19] Verborgh R, Steiner T, Van Deursen D, De Roo J, Van de Walle R, Vallés JG (2011). Description and interaction of restful services for automatic discovery and execution. *FTRA International Workshop on Advanced Future Multimedia Services*.

[20] Verborgh R, Steiner T, Van Deursen D, Van De Walle R, Valles JG Efficient runtime service discovery and consumption with hyperlinked RESTdesc.

[21] Berners-Lee T, Connolly D Notation3 (n3): a readable rdf syntax. http://www.w3.org/TeamSubmission/n3/.

[22] Nottingham M (2010). *Web Linking*.

[23] Kopecký J, Vitvar T, Bournez C, Farrell J (2007). SAWSDL: semantic annotations for WSDL and XML schema. *IEEE Internet Computing*.

[24] Martin D, Burstein M, Hobbs J, Lassila O http://www.w3.org/Submission/OWL-S/.

[25] Horrocks I, Patel-Schneider P, Boley H, Tabet S, Grosof B, Dean MS A semantic web rule language combining owl and ruleml. http://www.w3.org/Submission/SWRL/.

[26] D'Mello DA, Ananthanarayana V, Salian S (2011). A review of dynamic web service composition techniques. *Advanced Computing Communications in Computer and Information Science*.

[27] Verborgh R, Haerinck V, Steiner T Functional composition of sensor Web APIs.

